# Reducing dengue fever cases at the lowest budget: a constrained optimization approach applied to Thailand

**DOI:** 10.1186/s12889-021-10747-3

**Published:** 2021-04-27

**Authors:** Gerhart Knerer, Christine S. M. Currie, Sally C. Brailsford

**Affiliations:** 1 Mathematical Sciences, University of Southampton, Highfield, Southampton, SO17 1BJ UK; 2 Southampton Business School, University of Southampton, Highfield, Southampton, SO17 1BJ UK

**Keywords:** Dengue, Vaccination *Wolbachia*, Constrained optimization

## Abstract

**Background:**

With the challenges that dengue fever (DF) presents to healthcare systems and societies, public health officials must determine where best to allocate scarce resources and restricted budgets. Constrained optimization (CO) helps to address some of the acknowledged limitations of conventional health economic analyses and has typically been used to identify the optimal allocation of resources across interventions subject to a variety of constraints.

**Methods:**

A dynamic transmission model was developed to predict the number of dengue cases in Thailand at steady state. A CO was then applied to identify the optimal combination of interventions (release of *Wolbachia*-infected mosquitoes and paediatric vaccination) within the constraints of a fixed budget, set no higher than cost estimates of the current vector control programme, to minimize the number of dengue cases and disability-adjusted life years (DALYs) lost. Epidemiological, cost, and effectiveness data were informed by national data and the research literature. The time horizon was 10 years. Scenario analyses examined different disease management and intervention costs, budget constraints, vaccine efficacy, and optimization time horizon.

**Results:**

Under base-case budget constraints, the optimal coverage of the two interventions to minimize dengue incidence was predicted to be nearly equal (*Wolbachia* 50%; paediatric vaccination 49%) with corresponding coverages under lower bound (*Wolbachia* 54%; paediatric vaccination 10%) and upper bound (*Wolbachia* 67%; paediatric vaccination 100%) budget ceilings. Scenario analyses indicated that the most impactful situations related to the costs of *Wolbachia* and paediatric vaccination with decreases/ increases in costs of interventions demonstrating a direct correlation with coverage (increases/ decreases) of the respective control strategies under examination.

**Conclusions:**

Determining the best investment strategy for dengue control requires the identification of the optimal mix of interventions to implement in order to maximize public health outcomes, often under fixed budget constraints. A CO model was developed with the objective of minimizing dengue cases (and DALYs lost) over a 10-year time horizon, within the constraints of the estimated budgets for vector control in the absence of vaccination and *Wolbachia*. The model provides a tool for developing estimates of optimal coverage of combined dengue control strategies that minimize dengue burden at the lowest budget.

**Supplementary Information:**

The online version contains supplementary material available at 10.1186/s12889-021-10747-3.

## Background

Dengue fever (DF) is the most common vector-borne disease in Thailand as a result of rising incidence and increasing geographical incursion [1]. The main vectors of transmission for dengue in Thailand are the female mosquitoes of the *Aedes Aegypti* species and, to a lesser extent, *Aedes Albopictus*, with both prevalent in the country. All four of the dengue virus serotypes (DENV-1, DENV-2, DENV-3, DENV-4) circulate in Thailand [2] and have historically been associated with major dengue outbreaks in the country.

At present, the widespread prevention and control of DF is limited to the avoidance of mosquito bites and vector control measures, primarily based on insecticides and community engagement for environmental management initiatives [3]. Treatment consists primarily of supportive care, in the absence of licensed antiviral prophylactic or therapeutic treatments [4]. The dengue control strategy in Thailand is derived from World Health Organization (WHO) guidelines [3] consisting of three key elements: 1) avoiding transmission by preventing mosquito bites of people infected with dengue; 2) active community detection of non-consulting cases; and 3) vector control strategies comprising environmental management, source reduction, and chemical interventions (adulticide and/ or larvicide) [5].

With respect to dengue control by means of vaccination, only one dengue vaccine has been licensed, although uptake to date has been limited [6, 7], due in part to complex eligibility requirements amongst other factors [6]. A number of other dengue vaccines are under investigation, although at different stages of the development lifecycle with, for example, Phase 3 overall dengue vaccine efficacy results being recently published (and publicly presented) [8, 9].

In addition to the more traditional methods of vector control highlighted above, innovative ‘technologies’ are also undergoing evaluation, including the release of *Wolbachia* infection, which reduces the ability of *Aedes Aegypti* mosquitoes to transmit dengue, zika, chikungunya, and yellow fever [10, 11]. Female mosquitoes infected with the bacteria can pass this to their progeny and spread *Wolbachia* vertically across the generations. There is growing evidence of the effectiveness of large-scale deployments of *Wolbachia*-infected mosquitoes across different geographies resulting in substantive decreases in dengue incidence [12–14].

With the challenges that DF poses to healthcare systems and society at large, public health officials must determine where to allocate scarce resources to manage these problems and response(s). Cost-effectiveness analysis (CEA) is often used for healthcare resource allocation with the optimal allocation of resources achieved by selecting interventions in increasing order of their incremental cost-effectiveness ratios [15]. In a companion piece to the current study [16], a CEA was carried out to assess the impact of different control interventions in Thailand, focusing primarily on historical forms of vector control, but also anticipating new control strategies in the form of vaccination against dengue and *Wolbachia*-infected mosquitoes.

The emphasis of such analyses is on value for money, i.e. whether interventions are worth the ensuing investment, rather than who pays for it. Accordingly, CEAs highlight what decision-makers ideally should do, but not necessarily what they are practically able to do (within the potential budget available). As Sendi et al. [17] indicate, ‘ … decision-makers are increasingly constrained by a fixed-budget and may not be able to fund new more expensive interventions, even if they have been shown to represent good value for money’. CEA does not directly address this challenge, with commentators asserting that for local decision-makers, the criterion for determining how to spend public money (in the form of CEA) should be associated with the budget available for allocation [18].

Constrained optimization (CO) in the field of operational research (OR) assists in addressing some of the limitations of conventional health economic analyses and has been used to identify the optimal allocation of resources across interventions subject to a variety of constraints [19–21]. In two position papers, the International Society for Pharmacoeconomics and Outcomes Research Optimization Methods Good Practices Task Force underlined the facility of CO methods in healthcare when resources are constrained [22, 23]. Historically, OR methodologies have successfully been employed in a variety of optimization problems arising in healthcare [24–26]. In the field of infectious diseases, Brandeau [27–34] highlighted how OR-based models can help determine resource allocation that maximizes health benefits, providing important input into decision-making processes. In a similar vein, the identification and evaluation of optimal strategies to minimize infectious disease (subject to constraints) has also been explored by other authors by means of mathematical models, for example, in the determination of the most effective combination of preventive interventions for malaria [35, 36], human papillomavirus infection and cervical cancer [37, 38], and DF [39–42], amongst others [43, 44].

In this paper, we take up where the previous analyses, focused on CEA, concluded [16]. The CO approach applied in this study endeavours to provide decision-makers/ stakeholders with additional practical information when a proposed budget constraint is explicitly considered. The objective is not to make recommendations concerning specific control frameworks and/ or practical implementation for Thailand; rather, as highlighted, to complement CEA evidence as well as provide further insights into prioritizing and combining dengue control strategies.

The paper is organized as follows. First, we present a mathematical model of DF transmission with vaccination and *Wolbachia* as control interventions, economically assess the strategies under examination and propose a CO problem, the aim being to identify the optimal combination of these two interventions, within the constraint of a fixed budget, to minimize the number of dengue cases compared to steady state. We conclude with a discussion and next steps.

## Methods

### Resource allocation for infectious disease management

#### Objective function

Two separate and complimentary objective functions were used, namely, number of dengue cases (i.e. incident number of DF cases) and disability-adjusted life years (DALYs) lost. Number of dengue cases formed the primary objective function in base-case analyses, with DALYs lost as secondary.

The impact of interventions (including cumulative costs and effects) was estimated over a 10-year time horizon following intervention initiation. This follow-up period is believed to correspond to a reasonable timescale for public health decision-makers [45, 46].

#### Decision variables

*Vaccination:* acts on susceptible individuals with outputs governed by the balance between vaccine efficacy, vaccination coverage, and waning of protection. Similar to Knerer et al. [16], we used a dengue vaccine profile approximately consistent with (dengue) vaccines in late stage development and applied certain assumptions in this regard. The vaccine was assumed to have an overall protective efficacy of 73% (50 and 80% examined in scenario analysis) in all populations and against all grades of DF and an assumed duration of protection of 10 years. Additionally, it was assumed that the vaccine is effective after a course of vaccination, protects both seronegatives and seropositives, and has no adverse events or serious adverse events (breakthrough cases). Consistent with analyses undertaken in previous studies [16, 47], it is assumed that dengue vaccination would form part of routine paediatric vaccination and fit into existing child immunization schedules at age 1 year and under (in the current model, vaccination is administered at birth). When considering vaccination in combination with *Wolbachia*, it was assumed that vaccination coverage had arrived at steady state with no delay in implementation, i.e. there was no ramp-up period.

*Wolbachia:* This is a potential intervention for arbovirus control, demonstrating the ability to circulate amongst wild *Aedes aegypti* populations in field trials [48, 49] and with applications to chikungunya and zika virus as well as to DF, which share the same vector of transmission [50]. Potential outcomes of *Wolbachia* infection may include reduced egg-laying rates, reduced mosquito population, shorter (mosquito) lifespan and reduced transmission capabilities, which can greatly decrease the potential to spread mosquito-borne viral diseases (such as referred to above). A *Wolbachia* replacement strategy and mechanism of action involves the release of *Wolbachia*-infected mosquitoes into the natural mosquito environment, which subsequently mix and breed with native wild mosquitoes. *Wolbachia* infection takes place during reproduction resulting in the transformation of wild-type mosquito environments into *Wolbachia-*infected environments as the process replicates itself over generations of mosquitoes. Researchers have captured relevant differences between mosquitoes (*Wolbachia*-infected/ non-*Wolbachia-*infected) both explicitly (i.e. modelling *Wolbachia-*infected mosquitoes) and/ or implicitly (i.e. focusing on parameters affected by *Wolbachia*) in assorted models of differing complexity (e.g. Dorigatti et al. [51], Ndii et al. [52], Xue et al. [53], Shen [54], Bañuelos et al. [55], O’Reilly et al. [56]). Scaling factors are variously used to reflect evidence of, for example, changes in birth/ reproduction/ maturation rates (from aquatic to adult mosquito stage), mortality and biting rates, and human vector transmissibility [52, 53] due to *Wolbachia* infection. In this regard, mortality rates of *Wolbachia*-infected mosquitoes (wMel strain) are higher than non-*Wolbachia* vectors, as evidence shows that *Wolbachia* infection reduces the mosquito lifespan [51–54]. Similarly, *Wolbachia* infection is thought to hinder mosquito feeding and decrease the (successful) biting rate [52, 53] due to a condition known as bendy proboscis. In turn, a reduced biting rate also means that the overall human-to-vector transmission rate is reduced, as some *Wolbachia*-infected mosquitoes may not be infected with dengue virus due to a process known as ‘viral replication inhibition’ [52, 53, 55].

Given the somewhat exploratory nature of these analyses, we made a number of simplifying assumptions and compared long-term epidemiological projections with another study [51] as a basic validation check. In the previous analysis, dengue disease was suppressed for approximately 25 years before any meaningful rebound in incidence was observed [51]. We focused only on the situation where *Wolbachia*-infected mosquitoes arrive to steady-state/ fixation in the (mosquito) population after a period of release and the possibility to reduce or eliminate the disease in the human population. Accordingly, factors such as the necessary and sufficient conditions for *Wolbachia* penetration and propagation in the *Aedes aegypti* population or optimal release strategy are not considered. Model parameters impacted by *Wolbachia* infection, including mosquito death and biting rates, and transmissibility of infection, are modified (using scaling factor estimates derived from the literature), to convert non-*Wolbachia* parameters to *Wolbachia*-infected parameters [52, 53]. The scaling factors used in the analysis are presented in Table 1. In a previous study by the authors [16], a model-based analysis estimated a country wide *Wolbachia* release programme in Thailand would result in a decrease of approximately 84% in disease burden over 10 years (using the same scaling factors referred to above). This is broadly consistent with estimates from the literature referenced above as well as a model-based analysis predicting that a nationwide *Wolbachia* replacement programme instigated in Indonesia (100% coverage) would prevent approximately 86% of cases in the longer term [56].

**Table 1:** Parameter notation, values, and sources

Symbol	Definition	Value	Data source
*μ*_*h*_	Human birth rate = death rate	1/(70 × 365)	[47]
*μ*_*v*_	Vector mortality rate (non-*Wolbachia*)	12 days^− 1^	[57]
*Τ*_*v*_	Average extrinsic incubation rate	9 days^− 1^	[57]
*Τ*_*h*_	Average intrinsic incubation rate	7 days^− 1^	[57]
ɣ_*h*_	Human recovery rate	6 days^− 1^	[58]
*β*_*hv*_	Transmission probability, vector (non-*Wolbachia*) to host	0.186	Modelled
*β*_*vh*_	Transmission probability, host to vector (non-*Wolbachia*)	0.186	Modelled
*b*_*v*_	Biting rate (non-*Wolbachia*)	[0, 1]	[57]
*ε*	Vaccine efficacy	73%	Assumed^a^
θ	Waning rate at which temporarily protected individuals with dengue vaccine become partly susceptible to DF	10 years	Assumed
p	Proportion (coverage) of population vaccinated at birth	[0, 1]	Modelled
*μ*_*w*_	Vector mortality rate (*Wolbachia*)	1.10 × μ_v_	[52, 53]
*Τ*_*w*_	Average extrinsic incubation rate (*Wolbachia*)	*Τ*_*v*_	[52, 53]
*b*_*w*_	Biting rate (*Wolbachia*)	0.95 × b_v_	[52, 53]
*β*_*hw*_	Transmission probability, vector (*Wolbachia*) to host	0.5 × *β*_*hv*_	[52, 53]
*β*_*wh*_	Transmission probability, host to vector (*Wolbachia*)	*Β*_*vh*_	[52, 53]
*Br*	Scaling factor, vector birth rate (*Wolbachia*)	0.95	[52, 53]
*W*	*Wolbachia* release coverage	[0, 1]	Modelled

*DF* dengue fever

^a^Informed by candidate vaccines in development [8, 9]

#### Budget constraints

The purpose of the budget constraint(s) is to approximate real-life settings, where decisions are formulated within a limited budget and very high levels of both vaccination and *Wolbachia* are unlikely to be fully realized. In the current context, the overall (available) budget was constrained to be no higher than cost estimate**s** of the current vector control programme.

Cost estimates of vector control of $0.396, $0.66, and $1.056 per capita per year for sustained vector control in Thailand, representing lower bound, base case, and upper bound estimates, respectively, were derived from Fitzpatrick et al. [59]. This equates to (discounted) budget constraints of approximately $251, $368, and $589 million (2013 United States Dollars) for lower bound, base case, and upper bound estimates respectively, for Thailand over 10 years.

#### Optimization routine

Simulation output suggests that the output surface for each of the objective functions is an inclined plane, with a small amount of curvature. As a result, we opted to perform a grid search to identify the best combinations of interventions to use that satisfy the budgetary constraints. As the search space is relatively low-dimensional and the simulation model runs moderately quickly, this is a reasonably efficient method for identifying the best mix. If the number of decision variables were to increase, more sophisticated optimization methods would be required.

In the grid search, the parameter space of the respective interventions (i.e. vaccination coverage 0–100% and *Wolbachia* [release] coverage 0–100%) is divided by 100 and then 10,000 simulations (i.e. 100 × 100) are run. The programme eliminates all combinations that exceed the pre-specified budget constraint and retains only those permutations that fall within the programme scope. The process then concludes with the presentation of the optimal combination of the two interventions that minimize the number of dengue cases and DALYs (subject to budget constraints).

#### Dynamic transmission model background

We modelled the transmission of DF in the population of Thailand, using a system of ordinary differential equations adapted and simplified from Knerer et al. [16, 47]. In the earlier studies, an age-structured susceptible–exposed–infectious–recovered/susceptible–exposed–infectious dynamic transmission model combining seasonality, consecutive infection by all four serotypes, cross-protection, and immune enhancement, as well as combined vector-host transmission was developed. The model was used to represent dengue transmission dynamics using parameters appropriate for Thailand and to assess the impact and cost-effectiveness of combined vector-control and vaccination strategies on disease dynamics.

In the current study, we do not model population age structure and assume only one ‘global’ dengue serotype is circulating, as the use of a single serotype/ infection model was considered sufficient to answer the research question under investigation and adhere to the principle of parsimony. The human population is divided into four compartments comprising: humans susceptible to dengue infection (S_h_), exposed to infection (E_h_), infected and infectious (I_h_), and recovered (R_h_) compartments. The total human population (N_h_) is equal to the sum of the populations of humans in all human compartments, i.e. N_h_ = S_h_ + E_h_ + I_h_ + R_h_. The life cycle of the mosquito is represented by three infection phases, susceptible mosquitoes (vectors) (S_v_), exposed (incubating) mosquitoes (E_v_), and infected and infectious mosquitoes (I_v_). The total vector (mosquito) population is equal to N_v_ (i.e. N_v_ = S_v_ + E_v_ + I_v_). The complete model without study interventions is presented in the system of equations below:

$$ \frac{d{S}_h}{dt}={\mu}_h{N}_h-\left({b}_v{\beta}_{hv}\frac{I_v}{N_h}\right){S}_h-{\mu}_h{S}_h+\theta {V}_h $$

$$ \frac{d{E}_h}{dt}=\left({b}_v{\beta}_{hv}\frac{I_v}{N_h}\right){S}_h-\left({\mu}_h+{\tau}_h\right){E}_h $$

$$ \frac{d{I}_h}{dt}={\tau}_h{E}_h-\left({\mu}_h+{\gamma}_h\right){I}_h $$

$$ \frac{d{R}_h}{dt}={\gamma}_h{I}_h-{\mu}_h{R}_h $$

$$ \frac{d{S}_v}{dt}={\mu}_v{N}_v-\left({b}_v{\beta}_{vh}{S}_v\frac{I_h}{N_h}\right)-{\mu}_v{S}_v $$

$$ \frac{d{E}_v}{dt}=\left({b}_v{\beta}_{vh}{S}_v\frac{I_h}{N_h}\right)-\left({\mu}_v+{\tau}_v\right){E}_v $$

$$ \frac{d{I}_v}{dt}={\tau}_v{E}_v-{\mu}_v{I}_v $$

In the presence of *Wolbachia*, the model is extended to include a *Wolbachia*-carrying mosquito population. The population of *Wolbachia*-carrying mosquitoes is divided into subpopulations of susceptible (S_w_), exposed (E_w_), and infectious (I_w_) mosquitoes, where S_w_ + E_w_ + I_w_ = N_w_. In total, the model comprises 11 compartments; four for the human population, three each for the two mosquito populations, and one for vaccination. The complete model with study interventions is presented in the system of equations below:

$$ \frac{d{S}_h}{dt}=\left(1-\varepsilon p\right){\mu}_h{N}_h-\left(\left({b}_v{\beta}_{hv}\frac{I_v}{N_h}\right)+\left({b}_w{\beta}_{hw}\frac{I_w}{N_h}\right)\right){S}_h-{\mu}_h{S}_h+\theta {V}_h $$

$$ \frac{d{V}_h}{dt}=\left(\varepsilon p\right){\mu}_h{N}_h-\left({\mu}_h+\theta \right){V}_h $$

$$ \frac{d{E}_h}{dt}=\left(\left({b}_v{\beta}_{hv}\frac{I_v}{N_h}\right)+\left({b}_w{\beta}_{hw}\frac{I_w}{N_h}\right)\right){S}_h-\left({\mu}_h+{\tau}_h\right){E}_h $$

$$ \frac{d{I}_h}{dt}={\tau}_h{E}_h-\left({\mu}_h+{\gamma}_h\right){I}_h $$

$$ \frac{d{R}_h}{dt}={\gamma}_h{I}_h-{\mu}_h{R}_h $$

$$ \frac{d{S}_v}{dt}={\mu}_v\left(1-W\right){N}_v-\left({b}_v{\beta}_{vh}{S}_v\frac{I_h}{N_h}\right)-{\mu}_v{S}_v $$

$$ \frac{d{E}_v}{dt}=\left({b}_v{\beta}_{vh}{S}_v\frac{I_h}{N_h}\right)-\left({\mu}_v+{\tau}_v\right){E}_v $$

$$ \frac{d{I}_v}{dt}={\tau}_v{E}_v-{\mu}_v{I}_v $$

$$ \frac{d{S}_w}{dt}={\mu}_wW{N}_w Br-\left({b}_w{\beta}_{wh}{S}_w\frac{I_h}{N_h}\right)-{\mu}_w{S}_w $$

$$ \frac{d{E}_w}{dt}=\left({b}_w{\beta}_{wh}{S}_w\frac{I_h}{N_h}\right)-\left({\mu}_w+{\tau}_w\right){E}_w $$

$$ \frac{d{I}_w}{dt}={\tau}_w{E}_w-{\mu}_w{I}_w $$

Initial conditions were derived by running the model to equilibrium steady state without any control interventions. Key model assumptions are as follows:

- The total human population (N_h_) is treated as constant, i.e. births balance deaths at rate μ_h_ with no immigration of infected individuals into the human populace.
- The mortality rate due to DF is assumed to be negligible (< 1% with appropriate medical care [6]) and is therefore not included in the model.
- The population is homogeneous, which means that every individual in a compartment is homogenously mixed with the other individuals.
- Mosquito bites are homogeneously distributed amongst all human hosts, which means that each mosquito can bite any human host with equal probability.
- There is no natural protection, i.e. humans and mosquitoes are assumed to be born susceptible and losses of immunity are not considered, nor are maternally derived antibodies.
- The mosquito has no resistant phase due to its relatively short life expectancy.
- The coefficient of transmission of the disease is fixed and does not vary seasonally in the base case.

Table 1 lists the parameter values and their units and sources.

#### Data, expansion factors, and calibration

Similar to Knerer et al. [16], epidemiological data from National Epidemiological Surveillance in Thailand [60–64] was used to populate the dynamic transmission model. For the years 2008–2012, there was an average of 82,505 reported cases of dengue per year, including 43,890, 1688 and 36,927 dengue haemorrhagic fever (DHF), dengue shock syndrome (DSS), and DF cases, respectively [60–64]. Approximately 74% of these cases were hospitalized (61,465), with 88 deaths per year (72% due to DSS, with the remainder attributable to DHF).

The average number of reported cases was adjusted by an expansion factor of 8.5 to derive total ‘actual’ dengue cases. This is consistent with suggested expansion factors in South-East Asia for converting total reported dengue cases into estimated ‘actual’ cases, ranging from approximately 3.8 in Malaysia, to 8.5 in Thailand and 19 in East Timor [65]. Similarly, expansion factors were also calculated for individual countries based on the active phase of the CYD14 trial, which varied according to case definitions (different laboratory or clinical criteria) [66]. For Thailand, these were 12.0, 8.6, and 8.8 for virologically confirmed dengue, clinically diagnosed and virologically confirmed dengue, and clinically diagnosed dengue, respectively [66].

Model estimates were calibrated with figures reported by the National Epidemiological Surveillance in Thailand in 2008–2012 [60–64] multiplied by an expansion factor to adjust for under-reporting. The transmission parameters for human (*β*_*hv*_) and vector(*β*_*vh*_) were calibrated using a gradient-based optimization loop that minimized the mean-square difference between the model and recorded observations (adjusted for under-reporting). Model code was written in MATLAB and the optimization function ‘*fminsearch’* was used. At steady state, the model predicted an average of approximately 697,000 dengue cases per year in Thailand for all age groups combined. This compares to the average number of reported DF/ DHF cases in Thailand for the period 2008–2012 [60–64] adjusted for underreporting [65], all age groups combined (*n* = 701,256), which indicates a good fit between observed and predicted data.

### Outcomes

DALY estimates were taken from Knerer et al. [16], which were calculated using the methodology described by Murray [67, 68]. In the former study, and consistent with the approach of Clark et al. [69], the authors assumed that unreported cases are likely less severe than reported cases, although may still hinder usual daily activities, but for a shorter length of time. Accordingly, similar disability weights had been assigned for both unreported and reported cases of DF, but for a shorter duration of time (4 and 10 days for unreported and reported cases, respectively).

### Costs

As with the outcomes described above, we derived disease as well as intervention costs from our earlier paper [16] and highlight salient details in the following sections.

In brief, unit costs (per DF episode) derived from Shepard et al. [70] were used to calculate the following costs:

i. Payer perspective:
  direct medical costs for inpatient and outpatient dengue cases.
ii. Societal perspective:
  direct medical costs for inpatient and outpatient dengue cases
  direct non-medical costs for inpatient and outpatient dengue cases
  indirect costs for inpatient and outpatient dengue cases.

Total costs are comprised of direct medical costs and intervention costs (detailed below) from the payer perspective; and direct medical costs, direct non-medical costs, and indirect costs, in addition to intervention costs, from the societal perspective.

Studies with applicable unit costs [71, 72] and used by other researchers – for example, Lee et al. [73] – were similarly not considered in the present study, for the reasons outlined in Knerer et al [16]. Namely, their reliance on expert opinion, secondary data, or being considered somewhat outdated, leading to potential under-estimation of costs [70].

Cost inputs and other values are presented in Table 2. As part of scenario analyses, an alternative unit cost profile (Fitzpatrick et al. [59]) was substituted to determine the impact on the base-case results.

**Table 2:** Base case and scenario analysis values and sources

Input	Base case	Scenario analysis
Vaccination target population	Paediatric population vaccinated at birth (0–100% coverage)	Paediatric population vaccinated at birth (70–100% coverage)
Optimization time horizon	10 years	5 years
Vaccine efficacy	73%	50%, 80%
Time horizon	10 years	5 years
Inpatient costs	- $266 DF inpatient direct medical costs [74] - $566.43 DHF inpatient direct medical costs [70] - $72.77 inpatient direct non-medical costs [70] - $54.59 inpatient indirect costs [70]	Unit cost profiles from Fitzpatrick et al. [59] - $141.55 hospital bed day, primary - $169.24 hospital bed day, specialist
Outpatient costs	- $141.61 outpatient direct medical costs [70] - $82.20 outpatient direct non-medical costs [70] - $13.65 outpatient indirect costs [70]	Unit cost profile from Fitzpatrick et al. [59] - $18.29 ambulatory clinic visit
Cost of ‘un-reported’ cases	$12.12 for clinic visit [73]	N/ A
Vaccine price per course	$40 plus $4 vaccine administration costs	$20 plus $4 vaccine administration costs; $60 plus $4 vaccine administration costs
*Wolbachia*	*Wolbachia* cost per dengue case averted of $1 (i.e. cost of release per person covered of $4.45)	*Wolbachia* cost per person covered of $1 [75–77]; *Wolbachia* cost per person covered of $15.05 [75–77] (adjusted to 2013 prices [78])

*DF* dengue fever, *DHF* dengue haemorrhagic fever

**Table 3:** Optimal combination of *Wolbachia* and paediatric dengue vaccination coverage to minimize the number of dengue cases (and DALYs lost) by budget constraint

Budget constraint ($ millions)	*Wolbachia* (%)	Paediatric vaccination (%)	Cases (millions)	DALYs lost	*Wolbachia* costs ($ millions)	Vaccination costs ($ millions)	Total costs (PP) ($ millions)
**Steady state**	**0**	**0**	**7.175**	**67,831**	**–**	**–**	**$337.830**^**a**^
≥ 590	100	100	1.022	9660	273.744	350.666	$678.674
≤ 589	67	100	1.046	9888	182.496	350.666	$588.677
≤ 368	50	49	1.194	11,288	135.489	170.020	$368.772
≤ 251	54	10	1.296	12,256	147.854	25.331	$251.601

Bold text indicates steady state estimates without control

*DALY* disability-adjusted life year, *PP* payer perspective

^a^$414 million from a societal perspective

#### Costs of unreported cases

Where costs were ascribed to unreported cases for type of treatment, it was assumed that any treatment costs for unreported cases were on an outpatient basis only (i.e. there were no hospitalizations and/ or deaths associated with unreported cases), in line with the likely less severe nature of these cases [16, 69]. Unreported hospitalizations and deaths have been documented and some estimations for hospitalizations exist for Thailand [70]. However, a conservative approach was employed in the estimation of these costs.

#### Productivity costs due to death

Any economic costs associated with premature mortality (i.e. productivity loss and lifetime earnings foregone) were not included in calculations due to concerns over the risk of double counting benefits associated with averted deaths [79, 80].

#### Intervention costs

In earlier cost-effectiveness analyses that also included exploratory analyses of the cost-effectiveness of large-scale deployment of *Wolbachia* infection [16], two different cost estimates were used to calculate the costs of a *Wolbachia* intervention (due to uncertainty in the costs of such an intervention): firstly, a *Wolbachia* cost per dengue case averted of $1 (which was then used to back-calculate a cost of release per person covered of $4.45) and secondly, a *Wolbachia* cost per person covered of $1 (the latter being an aspirational cost of the World Mosquito Programme *Wolbachia* method [75–77]).

In the current study and continuing with the exploratory nature of analyses, we use similar costs to those above, with a *Wolbachia* cost of $1 per dengue case averted being used in base-case analyses and a *Wolbachia* cost per person covered of $1 being examined in scenario analyses.

As an additional scenario analysis, we also use a cost per person covered of $15.05 (the mean of the accelerated costs in Brady et al. [78]) adjusted to 2013 prices (for consistency). This is the average of the cost per person for an accelerated *Wolbachia* programme ranging from approximately $12 to $21 per person. This includes both urban areas (~$12 per person covered) and rural areas (~$14–21 per person covered).

Costs were assigned over 4 years to simulate accelerated *Wolbachia* implementation to the point where *Wolbachia*-infected mosquitoes had reached steady state/ fixation in the population.

For vaccination, a cost of $40 per vaccination course and assumed vaccine administration costs of $4 was used [16].

### Discount rate

Costs were discounted at 3% per annum as suggested by Thailand’s Health Technology Assessment guidance and the WHO [81, 82].

### Scenario analyses

Scenario analyses were carried out on different features and input data of the model to test the robustness of simulated findings and identify key parameters of influence that may impact base-case findings. Analyses predominantly focused on different budget constraints, disease management costs (i.e. unit cost profile), intervention costs, vaccine efficacy, and time horizon. An additional scenario was examined in which the parameter search space for paediatric vaccination was restricted to 70–100% (rather than 0–100% in the base case). Table 2 details scenario analysis inputs and ranges.

**Table 4:** Scenario analyses: optimal combination of *Wolbachia* and paediatric dengue vaccination coverage to minimize the number of dengue cases (and DALYs lost) – base-case budget constraint

Scenario	*Wolbachia* (%)	Paediatric vaccination (%)	Cases (millions)	DALYs lost	Total costs (PP) ($millions)
*Wolbachia* cost (lower bounds)	100	71	1.073	10,143	368.820
Vaccine cost −50%	43	100	1.075	10,167	367.215
Unit cost profile [59]	45	64	1.161	10,979	368.916
80% vaccine efficacy	44	53	1.180	11,158	368.389
Societal perspective	51	43	1.206	11,399	368.846
Vaccine coverage 70–100%	22	70	1.216	11,500	368.680
50% vaccine efficacy	69	33	1.221	11,540	368.630
Vaccine cost + 50%	68	23	1.230	11,628	368.925
*Wolbachia* cost (upper bounds)	21	41	1.326	12,531	366.220
5-year follow-up	33	22	1.317	12,450	202.720

*DALY* disability-adjusted life year, *PP* payer perspective

## Results

At steady state, the simulation model predicted approximately 7 million symptomatic dengue cases (7.175 million) in Thailand for all age groups combined over a 10-year period. The estimated total DALYs lost in this period were approximately 67,831 with cumulative disease costs of $338 million from the payer perspective. In the following sections, we detail *Wolbachia* and vaccination coverage, dengue reductions, and associated costs (including disease and intervention costs) stratified by different budgetary constraints.

**Fig. 1:**
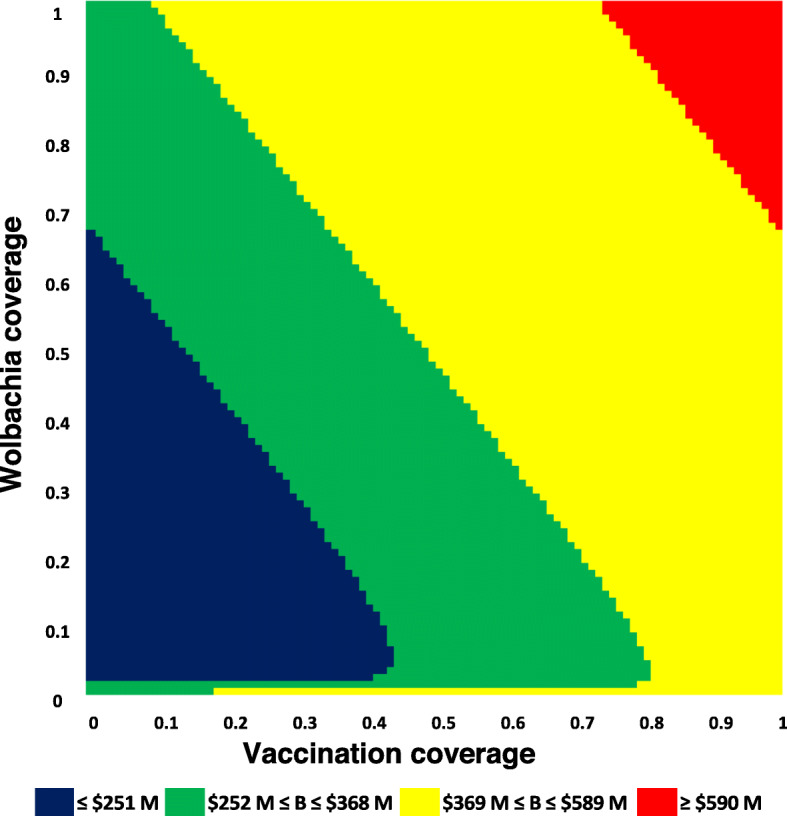
Heatmap of paediatric dengue vaccination coverage and *Wolbachia* coverage against budget constraints

In the unconstrained case, i.e., absence of budget restrictions or limits on investment (represented by the red section in Fig. 1), the projected optimal coverage of *Wolbachia* and paediatric vaccination (to minimize dengue incidence) comprised 100% coverage of each intervention. In this situation, a reduction of approximately 6 million dengue cases and 58,000 DALYs with an associated budget of $679 million, was forecast over 10 years (Table 3) versus steady state. *Wolbachia*-infected mosquito release costs of $274 million and vaccination costs of $351 million formed the great majority of the budget items. Table 3 also presents the optimal mix of the two interventions when budget constraints are introduced, encompassing base-case (approximately ≤ $368 million), lower bound (≤ $251 million), and upper bound (≤ $589 million) budget limits. Under base case budget constraints, the optimal coverage of the two interventions to minimize dengue cases (and DALYs lost) was predicted to be approximately even (*Wolbachia* 50%; paediatric vaccination 49%) although with different constituent costs (*Wolbachia* $135 million; vaccination $170 million). Corresponding intervention coverages estimated under lower and upper bound budgetary limits were *Wolbachia* 54% and paediatric vaccination 10% for the lower and *Wolbachia* 67% and paediatric vaccination 100% for the upper budget ceilings respectively. When resources become limited under the lowest budget constraints (≤ $251 million), *Wolbachia* has more impact on the population level of disease (as it becomes more affordable relative to vaccination cost) and vaccination is effectively reduced to a targeted hotspot control strategy.

Figure 1 presents a heat map of *Wolbachia* coverage and vaccination coverage against budget constraints. In this figure, intervention coverages are varied in the range 0–100% for each control strategy with the respective budget constraints colour coded ranging from the lowest budget constraint (≤ $251 million over 10 years) in blue to the absence of any budget constraint in red.

The heatmap illustrates, for example, the limits of intervention combinations by budgetary ceiling; the chart showing the highest possible combinations of *Wolbachia* and vaccination coverage that are feasible without exceeding upper limit budget constraints (as an example). In practical terms, this could take the form of either, for example, 100% *Wolbachia* coverage combined with approximately 74% paediatric vaccination coverage or approximately 67% *Wolbachia* coverage combined with 100% paediatric vaccination coverage. In effect, this suggests that one or other intervention can have very high coverage (i.e. 100%), but not both interventions without exceeding the budget ceiling. Similarly, from a more restricted budgetary standpoint (i.e. green section [base case] in Fig. 1), very high coverage of, for example, *Wolbachia*, is compatible with lower coverage of vaccination (or vice versa or midway for both), but high levels of coverage for both interventions together are not compatible within budget constraints. Depending on the public health goal, trade-offs may need to be determined to fulfil the desired objective. These trade-offs become less urgent as the budget available to fund interventions expands (as observed in the current study when the budget constraint is loosened). Additional file 1 shows dengue cases and DALYs lost by levels of *Wolbachia* and vaccination coverage.

When assessing the impact of alternative situations as part of wider scenario analyses (Table 4), approximately half of the scenarios display lower dengue incidence (and DALYs lost), with the remainder demonstrating greater incidence versus base-case projections (all within the budget ceiling of ≤ $368 million discounted over 10 years). The most impactful scenarios relate to the costs of *Wolbachia* and paediatric vaccination with decreases and/ or increases in costs of interventions demonstrating a direct correlation with the coverage (increases and/ or decreases) of the interventions under examination. For example, a reduction in vaccine acquisition costs results in a corresponding increase in paediatric vaccination coverage (100%) and a smaller reduction in *Wolbachia* coverage (i.e. more resources are directed to the lower-cost vaccination programme). Similarly, a decrease in *Wolbachia* costs gives rise to greater coverage (i.e. a large-scale countrywide *Wolbachia* release programme) as well as an increase in vaccination coverage (i.e. more resources are directed to vaccination). Conversely, as intervention costs increase, more investment flows to the less costly option and coverage increases as a result. When substituting a different and, in this case, lower unit cost profile [59], more funds are seemingly freed up for vaccination, with higher coverage compared to *Wolbachia,* reflecting the influence of unit costs in this regard. An increase in vaccine efficacy, from 73% to 80% representing the best case, results in greater resources being targeted towards vaccination and less to *Wolbachia (*although the change in coverage and resultant outcomes are relatively small). A decrease in vaccine efficacy, from 73% to 50% representing the worst case, results in the converse with resources directed more to *Wolbachia* and away from vaccination. When paediatric vaccination is restricted to the range 70–100% (in the grid search), left over investment above the minimum vaccination coverage of 70%, flows to *Wolbachia* (22%) and away from vaccination in order to optimally maximise public health outcomes at the lowest cost.

## Discussion

This study aimed to provide further insights into the prioritization and combination of dengue control strategies. The impact of *Wolbachia* infection (wMel strain) and vaccination on the dengue disease burden in Thailand was investigated as part of a constrained optimization problem. The primary goal of the exercise was to identify the best combination of vaccination and *Wolbachia* to minimize the number of dengue cases (and DALYs lost) subject to explicit budgetary constraints. We used a case study of Thailand for the analysis and set the budget constraint to be equal to the estimated current per capita spend on vector control in Thailand [59].

The paper acts as a complement to a CEA conducted by the same authors [16], which investigated both historical methods of dengue control as well as new technologies. For the most part, health economic model analyses are typically unconstrained, the assumption being that resources are available as needed and thus, affordable [22]. In practical terms, the reality may be that funding is absent, as programmes are frequently subject to national and local budget constraints. From a global perspective, many interventions remain under- or even un-funded by countries, although still falling within WHO cost-effectiveness thresholds and considered value for money as a result [18]. In low- and middle-income countries (and increasingly in more developed markets), other considerations beyond cost-effectiveness are likely important for decision-making, including affordability, overall budget impact and sustainability of funding amongst others [18].

The base epidemiological model underpinning the optimization analyses was shown to calibrate well at steady state with average reported symptomatic DF cases in Thailand for the years 2008–2012 [60–64], adjusted for under-reporting [16, 47, 65]. As a validation check, predictions were compared with previous model projections presented in Knerer et al. [16], derived using a different model structure and fitted to age-specific data on baseline dengue infection levels (2008–2012) [60–64]. Comparable figures for DALYs lost and cumulative disease costs over 10 years were approximately 67,595 DALYs lost and $336 and $412 million [16] from the payer and societal perspectives, respectively, indicating good concordance between the different model outputs.

Our results suggest that several different combinations of *Wolbachia* and vaccination (paediatric) can produce analogous reductions in the incidence of dengue cases yet have different budget impacts (comprising disease and intervention costs) to achieve the respective coverages. In the base case, the optimal mix between the two study interventions was shown to be approximately equivalent. Conversely, when an alternative (lower) unit cost profile [59] was substituted in scenario analyses, more resources were directed to vaccination with a resulting higher coverage than *Wolbachia*, reflecting the influence of unit costs in this regard. A priori hypotheses in relation to the optimal mix and cost of interventions were also borne out. For example, reduced *Wolbachia* costs would lead one to surmise a congruent increase in *Wolbachia* coverage (and decrease in vaccination coverage) whereas an increase in *Wolbachia* costs would lead to the opposite. Similarly, reductions/ increases in both vaccine acquisition costs, and efficacy would have parallel effects.

This study is subject to a number of important limitations. Similar to Knerer et al. [16], the transmission model used in this analysis does not account for asymptomatic cases, rather focussing on the economic impact of clinically apparent (symptomatic) cases and their remission. The vaccine profile employed in this study was informed by real-world overall efficacy data [8, 9]. For simplicity, a global serotype transmission model was used that does not explicitly account for individual serotypes (i.e. DENV-1, DENV-2, DENV-3, and DENV-4) nor the potential effects of secondary cases. Hence, any apparent reported imbalances in vaccine immune response between different serotypes and any potential negative implications that may follow from this were not considered. Regarding the use of reported efficacy data [8, 9], estimates were applied to the target population under the respective vaccination schedule in the study, rather than the age demographic specified in the original trial. The assumption was that any age-based recommendation would subsequently be extended to include younger children (including those under study). The reported overall vaccine efficacy was also assumed to be constant for the course of study follow-up (10 years) and therefore did not decrease over time. This may have led to possible overestimation in the base case of the impact of vaccination in the longer term. As a counterbalance, a much lower vaccine efficacy (50%) was examined in scenario analyses to reflect uncertainty in published overall vaccine efficacy results in relation to long-term waning of vaccine protection. Sensitivity analysis was restricted to those parameters that did not form part of the calibrated model. Epidemiological variables including biting rate, vector mortality rate, and transmission rate that were part of the calibrated model were therefore not examined. With respect to vector mortality rate, Ndii [83] reasoned that a maximum 10% reduction in *Wolbachia*-infected vector mortality rate (as used in the current analyses) was appropriate, citing evidence that an increased rate would result in the *Wolbachia*-infected mosquito population dying out and the non-*Wolbachia* infected population dominating the environment. Geographical specificity/ heterogeneity was also not considered in the analyses, but would perhaps be of value to help to characterize the optimal split between the two study interventions (to minimize dengue infection) at a finer spatial resolution, for example, north versus south, urban hotspots versus rural locations, etc. *Wolbachia* coverage will have additional benefits to the human population (beyond the dengue mitigation included in the current model), for example, in those areas of Thailand where there is a preponderance of chikungunya and/ or Zika virus. Whilst dengue is prevalent throughout Thailand, research on the long-term circulation of Zika virus indicates elevated risks of the disease (relative to the country as a whole) in the northeast and east of Thailand and reduced risks in the south of the country [84]. Conversely, an ongoing outbreak of chikungunya (since October 2018) indicates that cases are concentrated in Southern Thailand [85]. Historically, a large outbreak of Chikungunya in 2008–2010 was also located in the south of Thailand [86], reaching approximately a third of country districts with a subsequent sero-survey in 2014 confirming the extent of chikungunya penetration in this geographical area (estimated seroprevalence of approximately 29.6%) [87]. Notwithstanding the potential benefits of a spatial perspective to such analyses, this does not preclude additional sources of heterogeneity in the local setting, which may affect the feasibility of implementing different strategies and thus the overall results.

As previously highlighted in Knerer et al. [16], it is acknowledged that many practical hurdles still exist before a widespread *Wolbachia*-based dengue control strategy could be implemented. These include, for example, the optimal choice of *Wolbachia* strain, appropriate surveillance, and monitoring of environmental and evolutionary changes, as well as community ‘buy-in’ and acceptance [88, 89]. Certainly, the premise that is being examined in this study is not the ‘how’ of implementation, rather what the possible population impact could be once *Wolbachia*-infected mosquitoes have arrived at equilibrium/ steady state fixation in areas where they have been released. Although coverage is likely to be limited initially, such analyses provide insights into the human population impact of a potential *Wolbachia* programme on a large, countrywide scale, both separately and in combination with other control strategies.

## Conclusions

Our model provides a tool for developing estimates of optimal coverage of combined dengue control strategies (*Wolbachia* and paediatric vaccination) that minimize dengue burden at the lowest budget. If proposals/ suggestions are usefully to be put forward in relation to broader vaccine and/ or *Wolbachia* introduction for dengue control, policy and decision makers will likely need to determine which dengue interventions to prioritize to optimize the health status of the population, which may necessitate trade-offs depending on the public health goal. As alluded to above, practical operational realities may conceivably be more complicated than the somewhat simplified analyses presented here; in particular, the source of funding budgets for vaccination and/ or *Wolbachia* may be quite distinct, and thus not reflect the trade-offs discussed in this study. Notwithstanding this, commentators suggest that long-term dengue control necessitates increasing investment, complementary control strategies, and intervention programmes across a broad geographic area to minimize cross-border infection [90]. Accordingly, selecting the best investment strategy for dengue control requires the identification of the optimal mix of interventions to implement to maximize public health outcomes. This is often under fixed budgetary constraints and depends on the characteristics of the control strategies in each dengue setting. In this vein, important questions for future work and potential next steps include: (1) Should further investments in dengue interventions focus primarily on reinforcing existing control protocols and/ or increasing the coverage of current interventions and/ or introducing new ones (vector control tools and integrated strategies) and under what circumstances? (2) In what manner should a combination of interventions be further expanded to achieve specified public health objectives at the lowest budget (and potentially in the context of budget cuts in health)?

## Supplementary Information

**Additional file 1: Table S1.** Dengue cases (DALYs lost) by levels of *Wolbachia* and vaccination coverage

## Data Availability

The datasets used and/ or analysed during the current study are available from the corresponding author on reasonable request.

## Abbreviations

CEA: Cost-effectiveness analysis
CO: Constrained optimization
DALYs: Disability-adjusted life years
DF: Dengue fever
DHF: Dengue haemorrhagic fever
DSS: Dengue shock syndrome
E_h_: Humans exposed to dengue infection
E_v_: Exposed (incubating) mosquitoes (vectors)
E_w_: Exposed *Wolbachia*-carrying mosquitoes
I_h_: Dengue infected and infectious humans
I_v_: Infected and infectious mosquitoes (vectors)
I_w_: Infectious *Wolbachia*-carrying mosquitoes
N_h_: Total human population
OR: Operational research
R_h_: Humans recovered from dengue infection
S_h_: Humans susceptible to dengue infection
S_v_: Susceptible mosquitoes (vectors)
S_w_: Susceptible *Wolbachia*-carrying mosquitoes
WHO: World Health Organization
