# Supplementary material for: Reducing dengue fever cases at the lowest budget: a constrained optimization approach applied to Thailand
Source: BMC Public Health. 2021 Apr 27;21:807. doi: 10.1186/s12889-021-10747-3 (PMC8080389; doi:10.1186/s12889-021-10747-3)
Supplement: Supplementary file 1 — Additional file 1: Table S1. Dengue cases (DALYs lost) by levels of Wolbachia and vaccination coverage [file 12889_2021_10747_MOESM1_ESM.pdf]

Table S1. Dengue cases (DALYs lost) by levels of *Wolbachia* and vaccination coverage[a]

		Vaccination coverage (%)				
		20%	40%	60%	80%	100%
Wolbachia coverage (%)	20%	1,417,931 (13,405)	1,337,095 (12,641)	1,267,332 (11,981)	1,200,286 (11,347)	1,139,607 (10,774)
	40%	1,303,028 (12,319)	1,239,815 (11,721)	1,184,649 (11,199)	1,130,722 (10,690)	1,080,824 (10,218)
	60%	1,252,812 (11,844)	1,196,744 (11,314)	1,147,228 (10,846)	1,098,567 (10,386)	1,053,175 (9,957)
	80%	1,220,827 (11,541)	1,169,162 (11,053)	1,122,875 (10,615)	1,077,378 (10,185)	1,035,018 (9,785)
	100%	1,199,360 (11,339)	1,149,906 (11,339)	1,105,994 (10,456)	1,062,461 (10,044)	1,021,832 (9,660)

[a] Total costs from the payer perspective (comprised of direct medical costs and intervention costs) represented by colour coded categories as follows:

≤ $251 M **$252 M ≤ B ≤ $368 M** **$369 M ≤ B ≤ $589 M** **≥ $590 M**

DALYs, disability-adjusted life years.
